# Poly[tris­(2,5-dimethyl­benzene-1,4-­dicarboxyl­ato)bis­(pyridine)trizinc(II)]

**DOI:** 10.1107/S1600536810004848

**Published:** 2010-02-13

**Authors:** Fang-Kuo Wang, Shi-Yao Yang, Rong-Bin Huang, Li Nie

**Affiliations:** a Department of Chemistry, West Anhui University, Lu’ an 237012, People’s Republic of China; b Department of Chemistry, Xiamen University, Xiamen 361005, People’s Republic of China

## Abstract

The asymmetric unit of the title polymeric compound, [Zn_3_(C_10_H_8_O_4_)_3_(C_5_H_5_N)_2_]_*n*_ or [Zn_3_(dmbdc)_3_(py)_2_]_*n*_ (dmbdc = 2,5-dimethyl­benzene­dicarboxyl­ate; py = pyridine) contains two Zn(II) ions, one of which is located on an inversion centre, one and a half 2,5-dimethyl­benzene­dicarboxyl­ate ligands and one pyridine ligand. Each ZnO_6_ octa­hedron is sandwiched between two ZnO_4_N square-pyramids, forming a trinuclear zinc secondary building unit (SBU); each SBU is further linked by six 2,5-dimethyl­benzene­dicarboxyl­ate ligands with six adjacent trinuclear zinc SBU’s, forming a two-dimensional layer structure with a (3,6) net. One of the three zinc ions is octa­hedrally coordinated and the other two are square-pyramidally coordinated. The coordination modes for 2,5-dimethyl­benzene­dicarboxyl­ates are bis­(bidentate) or bidentate-tridentate.

## Related literature

For the potential applications of metal-organic frameworks formed from terephthalic acid and its derivatives, see Wang *et al.* (2007 ▶); Grzesiak *et al.* (2006 ▶); Rosi *et al.* (2005 ▶); Burrows *et al.* (2005 ▶); Liao *et al.* (2006 ▶); Yang *et al.* (2002 ▶); Eddaoudi *et al.* (2002 ▶). For related structures, see: Wang *et al.* (2008 ▶); Zhou *et al.* (2009 ▶).
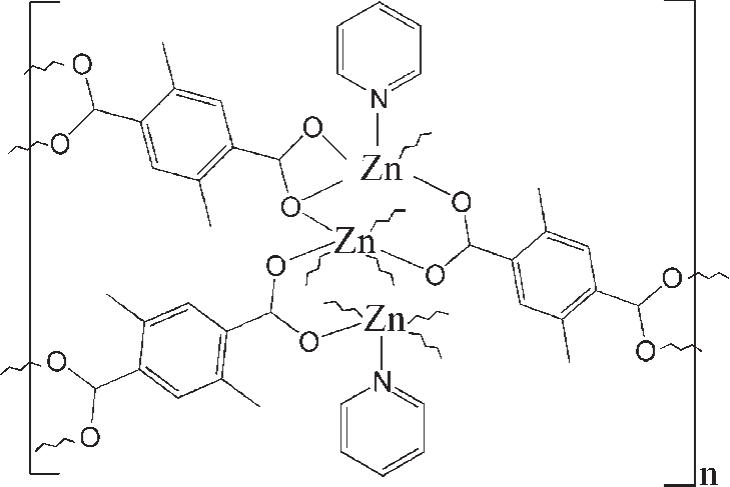


## Experimental

### 

#### Crystal data

[Zn_3_(C_10_H_8_O_4_)_3_(C_5_H_5_N)_2_]
*M* *_r_* = 930.80
Monoclinic, 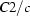

*a* = 22.3372 (15) Å
*b* = 10.2643 (7) Å
*c* = 16.9261 (11) Å
β = 105.140 (1)°
*V* = 3746.0 (4) Å^3^
*Z* = 4
Mo *K*α radiation
μ = 1.97 mm^−1^
*T* = 299 K
0.12 × 0.08 × 0.07 mm

#### Data collection

Bruker SMART APEX area-detector diffractometer
Absorption correction: multi-scan (*SADABS*; Bruker, 2002 ▶) *T*_min_ = 0.798, *T*_max_ = 0.874
16107 measured reflections
4487 independent reflections
3559 reflections with *I* > 2σ(*I*)
*R*_int_ = 0.059

#### Refinement

*R*[*F* ^2^ > 2σ(*F* ^2^)] = 0.066
*wR*(*F* ^2^) = 0.156
*S* = 1.08
4487 reflections
262 parameters
H-atom parameters constrained
Δρ_max_ = 0.74 e Å^−3^
Δρ_min_ = −0.63 e Å^−3^

### 

Data collection: *SMART* (Bruker, 2002 ▶); cell refinement: *SAINT* (Bruker, 2002 ▶); data reduction: *SAINT*; program(s) used to solve structure: *SHELXS97* (Sheldrick, 2008 ▶); program(s) used to refine structure: *SHELXL97* (Sheldrick, 2008 ▶); molecular graphics: *ORTEPII* (Johnson, 1976 ▶); software used to prepare material for publication: *SHELXL97*.

## Supplementary Material

Crystal structure: contains datablocks I. DOI: 10.1107/S1600536810004848/bv2132sup1.cif

Structure factors: contains datablocks I. DOI: 10.1107/S1600536810004848/bv2132Isup2.hkl

Additional supplementary materials:  crystallographic information; 3D view; checkCIF report

## Figures and Tables

**Fig. 1.**
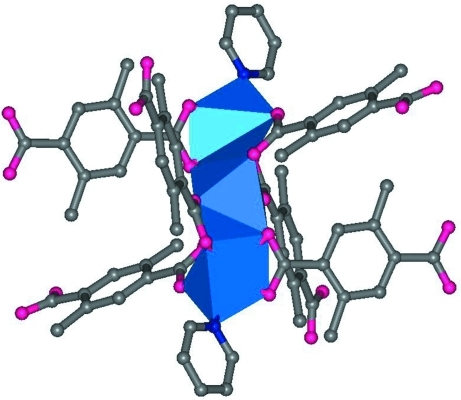
The connection of the trinuclear zinc SBU's. (Hydrogen atoms are omitted for clarity.)

**Fig. 2.**
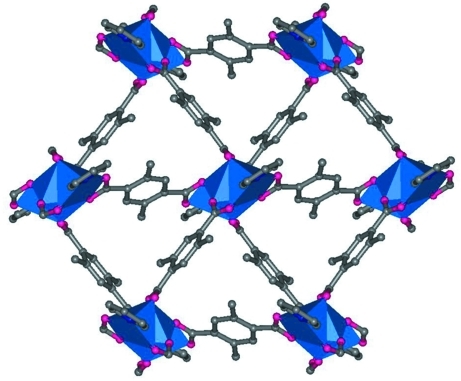
A perspective view of the two-dimensional (3, 6) net of 1 on bc plane. (Hydrogen atoms are omitted for clarity.)

**Fig. 3.**
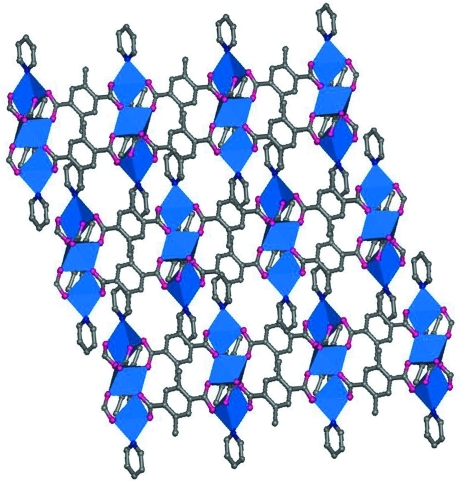
View of two-dimensional (3, 6) net of 1 stacked by ABAB mode along b axis. (Hydrogen atoms are omitted for clarity.)

## supplementary crystallographic information

### Comment

Terephthalic acid and its derivatives have been used to construct metal-organic frameworks. Some of these compounds display interesting structures and have potential applications (Wang *et al.*, 2007; Grzesiak *et al.*, 2006; Rosi *et al.*, 2005; Burrows *et al.*, 2005; Liao *et al.*, 2006; Yang *et al.*, 2002; Eddaoudi *et al.*,2002; Zhou *et al.*, 2009). In this paper we report a coordination polymer [Zn_3_(dmbdc)_3_(py)_2_]_n_, 1 (dmbdc=2,5-dimethylbenzenedicarboxylate; py=pyridine) synthesized by hydrothermal reaction.

The structure of 1 contains trinuclear zinc SBU's (SBU = Secondary Building Unit) (Fig. 1), in which each ZnO_6_ octahedron sandwiched between two ZnO_4_N square-pyramids. The three Zn ions exhibit two different coordination geometries: Zn1 coordinates to four oxygen atoms from three carboxylates in the plane and a nitrogen atom from py (py = pyridine) at the apex giving a distorted square-pyramidal geometry; Zn2 atom is coordinated by six oxygen atoms from six dmbdc anions (dmbdc=2,5-dimethylbenzenedicarboxylate) to constitute a slightly distorted octahedral environment. Coordination polymers with similar but different trinuclear zinc SBU's have been reported in recent years (Wang *et al.*, 2008). There are two coordination modes for the dmbdc in the structure of 1, one is bis(bidentate), and the other one adopts bidentate and tridentate for each of its carboxyl groups. However, each trinuclear zinc SBU is further linked by six dmbdc ligands to six adjacent trinuclear zinc SBU's to form a two-dimensional sheet with a (3,6) net parallel to *bc* plane (Fig. 2). The two-dimensional sheets are further packed in an ABAB··· mode along *b* axis (Fig. 3). Coordinated pyridine molecules lie in the both sides of trinuclear zinc SBU's respectively.

### Experimental

A suspension of 2,5-dimethylbenzenedicarboxylic acid (H_2_dmbdc, 0.097 g, 0.50 mmol) in H_2_O (12 ml), pyridine was slowly added to the solution until pH was adjusted to 7, then Zn(NO_3_)_2_^.^6H_2_O (0.15 g, 0.50 mmol) was added. The mixture was placed in a 20 ml Teflon-lined vessel, heated to 120°C at the rate of 0.2°C/min, and kept at 120°C for 3 days, then slowly cooled down to room temperature at the rate of 0.1°C/min. Colorless platelet crystals (0.062 g, yield 40%) were separated by filtration, washed with deionized water and dried in air. Elemental Analysis: C_40_H_34_N_2_O_12_Zn_3_, found (calc.) C 51.52 (51.61), H 3.66 (3.69), N 2.99 (3.01). FTIR (KBr, cm^-1^): 3454 (*m*), 3031 (w), 2961 (*m*), 2739 (w), 1922 (w), 1820 (*m*),1452 (*m*), 1399 (*s*), 1347 (*m*), 1158 (*m*), 1074 (*s*), 918 (*s*), 797 (*versus*).

### Refinement

The aromatic H atoms were generated geometrically (C—H 0.93 Å) and were allowed to ride on their parent atoms in the riding model approximations, with their temperature factors set to 1.2 times those of the parent atoms. The methyl H atoms were generated geometrically (C—H 0.96 Å) and were allowed to ride on their parent atoms in the riding model approximations, with their temperature factors set to 1.5 times those of the parent atoms.

### Crystal data

**Table:**

[Zn_3_(C_10_H_8_O_4_)_3_(C_5_H_5_N)_2_]	*F*(000) = 1896
*M**_r_* = 930.80	*D*_x_ = 1.650 Mg m^−^^3^
Monoclinic, *C*2/*c*	Mo *K*α radiation, λ = 0.71073 Å
Hall symbol: -C 2yc	Cell parameters from 2781 reflections
*a* = 22.3372 (15) Å	θ = 2.2–25.0°
*b* = 10.2643 (7) Å	µ = 1.97 mm^−^^1^
*c* = 16.9261 (11) Å	*T* = 299 K
β = 105.140 (1)°	Block, colorless
*V* = 3746.0 (4) Å^3^	0.12 × 0.08 × 0.07 mm
*Z* = 4	

### Data collection

**Table:**

Bruker SMART APEX area-detector diffractometer	4487 independent reflections
Radiation source: fine-focus sealed tube	3559 reflections with *I* > 2σ(*I*)
graphite	*R*_int_ = 0.059
φ and ω scan	θ_max_ = 28.7°, θ_min_ = 1.9°
Absorption correction: multi-scan (*SADABS*; Bruker, 2002)	*h* = −28→30
*T*_min_ = 0.798, *T*_max_ = 0.874	*k* = −13→13
16107 measured reflections	*l* = −21→22

### Refinement

**Table:**

Refinement on *F*^2^	Primary atom site location: structure-invariant direct methods
Least-squares matrix: full	Secondary atom site location: difference Fourier map
*R*[*F*^2^ > 2σ(*F*^2^)] = 0.066	Hydrogen site location: inferred from neighbouring sites
*wR*(*F*^2^) = 0.156	H-atom parameters constrained
*S* = 1.08	*w* = 1/[σ^2^(*F*_o_^2^) + (0.0282*P*)^2^ + 12.5564*P*] where *P* = (*F*_o_^2^ + 2*F*_c_^2^)/3
4487 reflections	(Δ/σ)_max_ < 0.001
262 parameters	Δρ_max_ = 0.74 e Å^−^^3^
0 restraints	Δρ_min_ = −0.62 e Å^−^^3^

### Special details

**Table:**

Geometry. All e.s.d.'s (except the e.s.d. in the dihedral angle between two l.s. planes) are estimated using the full covariance matrix. The cell e.s.d.'s are taken into account individually in the estimation of e.s.d.'s in distances, angles and torsion angles; correlations between e.s.d.'s in cell parameters are only used when they are defined by crystal symmetry. An approximate (isotropic) treatment of cell e.s.d.'s is used for estimating e.s.d.'s involving l.s. planes.
Refinement. Refinement of *F*^2^ against ALL reflections. The weighted *R*-factor *wR* and goodness of fit *S* are based on *F*^2^, conventional *R*-factors *R* are based on *F*, with *F* set to zero for negative *F*^2^. The threshold expression of *F*^2^ > σ(*F*^2^) is used only for calculating *R*-factors(gt) *etc*. and is not relevant to the choice of reflections for refinement. *R*-factors based on *F*^2^ are statistically about twice as large as those based on *F*, and *R*- factors based on ALL data will be even larger.

### Fractional atomic coordinates and isotropic or equivalent isotropic displacement parameters (Å^2^)

**Table:**

	*x*	*y*	*z*	*U*_iso_*/*U*_eq_	
Zn1	0.40381 (2)	0.67147 (5)	0.55736 (3)	0.02859 (17)	
Zn2	0.2500	0.7500	0.5000	0.02465 (19)	
O1	0.40247 (16)	0.7797 (3)	0.4622 (2)	0.0379 (8)	
O2	0.31369 (15)	0.8673 (3)	0.4686 (2)	0.0347 (7)	
O3	0.29594 (17)	1.2435 (4)	0.12228 (19)	0.0425 (9)	
O4	0.39737 (16)	1.2197 (4)	0.1478 (2)	0.0426 (9)	
O5	0.37531 (18)	0.4905 (4)	0.5713 (3)	0.0557 (11)	
O6	0.30025 (18)	0.5819 (3)	0.4821 (2)	0.0444 (9)	
N1	0.49686 (18)	0.6283 (4)	0.5907 (3)	0.0397 (10)	
C1	0.3588 (2)	0.9476 (5)	0.3687 (3)	0.0309 (10)	
C2	0.4095 (2)	0.9664 (5)	0.3361 (3)	0.0368 (11)	
C3	0.4018 (2)	1.0506 (5)	0.2701 (3)	0.0362 (11)	
H3A	0.4354	1.0645	0.2483	0.043*	
C4	0.3476 (2)	1.1143 (4)	0.2354 (3)	0.0297 (9)	
C5	0.2970 (2)	1.0983 (5)	0.2683 (3)	0.0310 (9)	
C6	0.3049 (2)	1.0147 (5)	0.3343 (3)	0.0325 (10)	
H6A	0.2716	1.0031	0.3570	0.039*	
C7	0.3581 (2)	0.8590 (4)	0.4383 (3)	0.0297 (9)	
C8	0.4722 (3)	0.9037 (8)	0.3691 (5)	0.074 (2)	
H8A	0.5009	0.9382	0.3409	0.111*	
H8B	0.4686	0.8112	0.3608	0.111*	
H8C	0.4871	0.9218	0.4265	0.111*	
C9	0.3457 (2)	1.1994 (4)	0.1631 (3)	0.0308 (10)	
C10	0.2367 (3)	1.1692 (6)	0.2392 (4)	0.0521 (15)	
H10A	0.2090	1.1424	0.2709	0.078*	
H10B	0.2187	1.1493	0.1825	0.078*	
H10C	0.2439	1.2613	0.2454	0.078*	
C11	0.3226 (2)	0.4861 (5)	0.5232 (3)	0.0400 (12)	
C12	0.2872 (2)	0.3605 (4)	0.5142 (3)	0.0322 (10)	
C13	0.2293 (2)	0.3618 (4)	0.4598 (3)	0.0363 (11)	
H13A	0.2154	0.4392	0.4327	0.044*	
C14	0.3094 (2)	0.2458 (5)	0.5566 (3)	0.0381 (11)	
C15	0.3713 (3)	0.2313 (6)	0.6163 (4)	0.0630 (18)	
H15A	0.3768	0.1427	0.6351	0.095*	
H15B	0.3738	0.2881	0.6621	0.095*	
H15C	0.4033	0.2537	0.5903	0.095*	
C16	0.5188 (3)	0.5242 (8)	0.6329 (6)	0.103 (4)	
H16A	0.4911	0.4643	0.6447	0.124*	
C17	0.5805 (4)	0.5003 (10)	0.6601 (8)	0.134 (5)	
H17A	0.5944	0.4256	0.6906	0.161*	
C18	0.6214 (3)	0.5840 (8)	0.6433 (6)	0.081 (2)	
H18A	0.6638	0.5700	0.6634	0.098*	
C19	0.6002 (3)	0.6877 (7)	0.5972 (4)	0.0612 (17)	
H19A	0.6274	0.7453	0.5821	0.073*	
C20	0.5379 (3)	0.7074 (6)	0.5725 (4)	0.0536 (15)	
H20A	0.5234	0.7808	0.5411	0.064*	

### Atomic displacement parameters (Å^2^)

**Table:**

	*U*^11^	*U*^22^	*U*^33^	*U*^12^	*U*^13^	*U*^23^
Zn1	0.0302 (3)	0.0257 (3)	0.0305 (3)	−0.0001 (2)	0.0090 (2)	0.0002 (2)
Zn2	0.0236 (4)	0.0256 (4)	0.0249 (3)	−0.0023 (3)	0.0067 (3)	−0.0003 (3)
O1	0.0374 (18)	0.0410 (19)	0.0380 (18)	0.0055 (15)	0.0149 (15)	0.0153 (15)
O2	0.0362 (18)	0.0304 (17)	0.0437 (19)	−0.0038 (14)	0.0213 (15)	0.0055 (14)
O3	0.042 (2)	0.060 (2)	0.0234 (15)	0.0153 (17)	0.0050 (15)	0.0087 (16)
O4	0.0374 (19)	0.053 (2)	0.0365 (18)	−0.0024 (16)	0.0089 (15)	0.0189 (16)
O5	0.042 (2)	0.035 (2)	0.087 (3)	−0.0140 (17)	0.011 (2)	−0.007 (2)
O6	0.065 (2)	0.0217 (16)	0.058 (2)	−0.0018 (16)	0.038 (2)	−0.0008 (16)
N1	0.028 (2)	0.037 (2)	0.052 (3)	0.0043 (17)	0.0073 (18)	0.002 (2)
C1	0.029 (2)	0.036 (2)	0.030 (2)	−0.0017 (18)	0.0097 (18)	0.0038 (19)
C2	0.031 (2)	0.040 (3)	0.044 (3)	0.004 (2)	0.018 (2)	0.009 (2)
C3	0.032 (2)	0.045 (3)	0.036 (2)	0.002 (2)	0.016 (2)	0.009 (2)
C4	0.030 (2)	0.031 (2)	0.026 (2)	−0.0008 (18)	0.0042 (18)	0.0016 (18)
C5	0.027 (2)	0.033 (2)	0.031 (2)	−0.0005 (18)	0.0057 (18)	0.0025 (19)
C6	0.029 (2)	0.035 (2)	0.037 (2)	0.0010 (19)	0.0134 (19)	0.004 (2)
C7	0.031 (2)	0.026 (2)	0.033 (2)	−0.0048 (18)	0.0113 (19)	0.0013 (18)
C8	0.041 (3)	0.100 (5)	0.092 (5)	0.031 (4)	0.037 (3)	0.060 (5)
C9	0.036 (3)	0.028 (2)	0.027 (2)	−0.0017 (19)	0.0062 (19)	−0.0019 (18)
C10	0.036 (3)	0.070 (4)	0.051 (3)	0.013 (3)	0.014 (2)	0.020 (3)
C11	0.045 (3)	0.027 (2)	0.055 (3)	−0.005 (2)	0.027 (3)	−0.011 (2)
C12	0.032 (2)	0.021 (2)	0.049 (3)	−0.0012 (17)	0.019 (2)	−0.0027 (19)
C13	0.034 (2)	0.023 (2)	0.053 (3)	0.0055 (18)	0.012 (2)	0.007 (2)
C14	0.033 (2)	0.031 (2)	0.050 (3)	0.0039 (19)	0.009 (2)	0.002 (2)
C15	0.042 (3)	0.049 (4)	0.084 (5)	0.002 (3)	−0.008 (3)	0.014 (3)
C16	0.047 (4)	0.083 (6)	0.166 (9)	0.003 (4)	0.006 (5)	0.071 (6)
C17	0.045 (4)	0.109 (7)	0.231 (13)	0.015 (5)	0.006 (6)	0.098 (9)
C18	0.037 (3)	0.081 (5)	0.121 (7)	0.012 (4)	0.010 (4)	−0.002 (5)
C19	0.038 (3)	0.078 (5)	0.069 (4)	−0.011 (3)	0.015 (3)	−0.014 (4)
C20	0.043 (3)	0.059 (4)	0.059 (4)	−0.006 (3)	0.014 (3)	0.007 (3)

### Geometric parameters (Å, °)

**Table:**

Zn1—O4^i^	1.931 (3)	C4—C9	1.496 (6)
Zn1—O1	1.950 (3)	C5—C6	1.384 (6)
Zn1—O5	1.998 (4)	C5—C10	1.495 (7)
Zn1—N1	2.055 (4)	C6—H6A	0.9300
Zn1—C11	2.588 (5)	C8—H8A	0.9600
Zn2—O2	2.037 (3)	C8—H8B	0.9600
Zn2—O2^ii^	2.037 (3)	C8—H8C	0.9600
Zn2—O3^iii^	2.057 (3)	C10—H10A	0.9600
Zn2—O3^i^	2.057 (3)	C10—H10B	0.9600
Zn2—O6	2.123 (3)	C10—H10C	0.9600
Zn2—O6^ii^	2.123 (3)	C11—C12	1.500 (6)
O1—C7	1.265 (6)	C12—C13	1.378 (7)
O2—C7	1.233 (5)	C12—C14	1.401 (7)
O3—C9	1.231 (6)	C13—C14^vi^	1.385 (7)
O3—Zn2^iv^	2.057 (3)	C13—H13A	0.9300
O4—C9	1.264 (6)	C14—C13^vi^	1.385 (7)
O4—Zn1^v^	1.931 (3)	C14—C15	1.492 (7)
O5—C11	1.245 (6)	C15—H15A	0.9600
O6—C11	1.233 (6)	C15—H15B	0.9600
N1—C16	1.308 (8)	C15—H15C	0.9600
N1—C20	1.320 (7)	C16—C17	1.356 (10)
C1—C6	1.378 (6)	C16—H16A	0.9300
C1—C2	1.395 (6)	C17—C18	1.337 (11)
C1—C7	1.492 (6)	C17—H17A	0.9300
C2—C3	1.387 (7)	C18—C19	1.332 (10)
C2—C8	1.511 (7)	C18—H18A	0.9300
C3—C4	1.367 (6)	C19—C20	1.359 (8)
C3—H3A	0.9300	C19—H19A	0.9300
C4—C5	1.393 (6)	C20—H20A	0.9300
			
O4^i^—Zn1—O1	109.70 (17)	O2—C7—C1	117.6 (4)
O4^i^—Zn1—O5	110.64 (18)	O1—C7—C1	118.4 (4)
O1—Zn1—O5	133.67 (17)	C2—C8—H8A	109.5
O4^i^—Zn1—N1	100.73 (17)	C2—C8—H8B	109.5
O1—Zn1—N1	98.40 (16)	H8A—C8—H8B	109.5
O5—Zn1—N1	95.60 (17)	C2—C8—H8C	109.5
O4^i^—Zn1—C11	114.20 (16)	H8A—C8—H8C	109.5
O1—Zn1—C11	112.05 (17)	H8B—C8—H8C	109.5
O5—Zn1—C11	27.91 (16)	O3—C9—O4	124.3 (4)
N1—Zn1—C11	120.16 (17)	O3—C9—C4	120.2 (4)
O2—Zn2—O2^ii^	180.00 (18)	O4—C9—C4	115.5 (4)
O2—Zn2—O3^iii^	87.46 (14)	C5—C10—H10A	109.5
O2^ii^—Zn2—O3^iii^	92.54 (14)	C5—C10—H10B	109.5
O2—Zn2—O3^i^	92.54 (14)	H10A—C10—H10B	109.5
O2^ii^—Zn2—O3^i^	87.46 (14)	C5—C10—H10C	109.5
O3^iii^—Zn2—O3^i^	180.000 (1)	H10A—C10—H10C	109.5
O2—Zn2—O6	90.67 (13)	H10B—C10—H10C	109.5
O2^ii^—Zn2—O6	89.33 (13)	O6—C11—O5	121.0 (5)
O3^iii^—Zn2—O6	88.47 (15)	O6—C11—C12	120.2 (5)
O3^i^—Zn2—O6	91.53 (15)	O5—C11—C12	118.8 (5)
O2—Zn2—O6^ii^	89.33 (13)	O6—C11—Zn1	72.3 (3)
O2^ii^—Zn2—O6^ii^	90.67 (13)	O5—C11—Zn1	48.7 (2)
O3^iii^—Zn2—O6^ii^	91.53 (15)	C12—C11—Zn1	167.2 (4)
O3^i^—Zn2—O6^ii^	88.47 (15)	C13—C12—C14	119.8 (4)
O6—Zn2—O6^ii^	180.0 (2)	C13—C12—C11	116.0 (4)
C7—O1—Zn1	118.2 (3)	C14—C12—C11	124.3 (5)
C7—O2—Zn2	139.4 (3)	C12—C13—C14^vi^	123.7 (4)
C9—O3—Zn2^iv^	135.7 (3)	C12—C13—H13A	118.2
C9—O4—Zn1^v^	121.2 (3)	C14^vi^—C13—H13A	118.2
C11—O5—Zn1	103.4 (3)	C13^vi^—C14—C12	116.6 (5)
C11—O6—Zn2	136.0 (3)	C13^vi^—C14—C15	118.4 (5)
C16—N1—C20	116.5 (5)	C12—C14—C15	125.0 (5)
C16—N1—Zn1	122.3 (4)	C14—C15—H15A	109.5
C20—N1—Zn1	121.2 (4)	C14—C15—H15B	109.5
C6—C1—C2	118.3 (4)	H15A—C15—H15B	109.5
C6—C1—C7	116.7 (4)	C14—C15—H15C	109.5
C2—C1—C7	125.1 (4)	H15A—C15—H15C	109.5
C3—C2—C1	117.6 (4)	H15B—C15—H15C	109.5
C3—C2—C8	118.1 (4)	N1—C16—C17	122.5 (7)
C1—C2—C8	124.4 (4)	N1—C16—H16A	118.8
C4—C3—C2	123.6 (4)	C17—C16—H16A	118.8
C4—C3—H3A	118.2	C18—C17—C16	120.0 (8)
C2—C3—H3A	118.2	C18—C17—H17A	120.0
C3—C4—C5	119.5 (4)	C16—C17—H17A	120.0
C3—C4—C9	117.6 (4)	C19—C18—C17	118.8 (7)
C5—C4—C9	122.9 (4)	C19—C18—H18A	120.6
C6—C5—C4	116.7 (4)	C17—C18—H18A	120.6
C6—C5—C10	118.8 (4)	C18—C19—C20	118.5 (7)
C4—C5—C10	124.5 (4)	C18—C19—H19A	120.7
C1—C6—C5	124.3 (4)	C20—C19—H19A	120.7
C1—C6—H6A	117.8	N1—C20—C19	123.7 (6)
C5—C6—H6A	117.8	N1—C20—H20A	118.2
O2—C7—O1	124.0 (4)	C19—C20—H20A	118.2

Symmetry codes: (i) *x*, −*y*+2, *z*+1/2; (ii) −*x*+1/2, −*y*+3/2, −*z*+1; (iii) −*x*+1/2, *y*−1/2, −*z*+1/2; (iv) −*x*+1/2, *y*+1/2, −*z*+1/2; (v) *x*, −*y*+2, *z*−1/2; (vi) −*x*+1/2, −*y*+1/2, −*z*+1.
